# Graduating in uncertain times: The impact of COVID‐19 on recent graduate career prospects, trajectories and outcomes

**DOI:** 10.1111/hequ.12415

**Published:** 2022-12-24

**Authors:** Michael Tomlinson, Florence Reedy, Damon Burg

**Affiliations:** ^1^ Southampton Education School University of Southampton Southampton UK; ^2^ Association of Graduate Careers Advisory Services Sheffield UK; ^3^ University of Bolton Bolton UK

**Keywords:** early careers transitions, economic shocks, graduates, policy, scarring

## Abstract

This article examines the impacts of the COVID‐19 pandemic on recent UK graduates' initial employment outcomes and how they experience the transition into a challenging labour market context. We draw on longitudinal survey and interview data, collected from recent graduates who had mainly graduated during the onset of the COVID‐19 pandemic in summer 2020 that examines graduate perception of the labour market, impacts on labour market entry impacts and early career progression and effects of periods of unemployment or under‐employment. The article shows some of the main impacts of the recent pandemic‐affected labour market, including: widespread concerns about job opportunities and employer support, the perceived employment impacts of the pandemic and early signs of scarring and labour market disorientation amongst those who were struggling to find employment of their choice. Such experiences are clearly intensified during the specific COVID‐19 context, but the policy implications they raise have wider relevance for supporting graduates during future periods of labour market volatility.

## 1 INTRODUCTION AND CONTEXT

The COVID‐19 pandemic has a potentially profound impact on those entering the labour market, in particular recent graduates, who are seeking a return from their investment in higher education (HE). The economic impacts of the pandemic are far‐reaching, with weakened and destabilised national labour markets characterised by precarious work and vacancy reduction. Evidence relating to the economic fall‐out of the 2008 recession showed widespread challenges amongst young people and those leaving education for the first time, including sustained unemployment, financial risk, reduced training and career learning opportunities and underutilisation of skills to name a few (Mayhew & Anand, 2020; Suleman & Figueireido, 2020). Those seeking first‐time employment during a recessionary period are more likely to be in employment not aligned to their qualification level, accepting lower wages and less attractive working conditions than they might in more favourable economic conditions. In recessionary economic climates time‐honoured distinctions between ‘youth’ and ‘graduate’ labour markets become further blurred as graduates compete more intensely for suitable employment, increasingly with non‐graduates. HE qualifications function less efficiently as an occupational filtering mechanism in allocating people to occupations at different level of skills distribution (Lauder & Mayhew, 2020).

The COVID‐19 pandemic has been understood as an economic ‘shock event’ (Akkermans et al., 2020) which essentially destabilises otherwise clearer career pathways and forces individuals to reappraise their place in the labour market and how they may carve out sustainable career outcomes. This can either be transient or result in longer‐lasting scarring effects that deplete individuals' capacity to (re)integrate into the labour market. As an external global event outside of individuals' immediate control, the pandemic differentially impacts individuals depending on their life circumstance, location in the social structure and access to resources that may mitigate its most adverse effects. Evidence indicated that even early into the pandemic, the following economic fallout was unequally shouldered by people aged 18–24, those from an ethnic minority group, women, young workers and disabled workers (Powell et al., 2022).

One of the challenges in analysing the impact of the COVID‐19 pandemic on recent school and college leavers is separating the effects of a major economic shock such as the COVID‐19 pandemic from wider structural challenges that have preceded this over the past several decades. The labour market repercussions from the COVID‐19 pandemic have a bearing on both recent graduates (ONS, 2021) and those who have been the in the labour market for some time (Future Track, 2021), particularly those who have embarked on self‐employment or followed careers in sectors most at risk from national lockdown measures. Understanding the COVID‐19 pandemic as a temporary phenomenon, whose immediate impacts will be endured through economic and personal resilience as the economy recovers globally, elides two main realities. First is the speed and shape of recovery which may depend on the level by which the pandemic halts and normal full‐scale economic activity resumes. Economists have observed that this is unlikely to represent a neat recover shape (the so‐called ‘V‐shape’) and instead unevenness by which different sectors and markets improve (OECD, 2021). The current labour market represents a significant challenge for graduates, as they are transiting into it during a challenging economic period. Predictions suggest that the UK economy is likely to contract again, resulting in a further long period of recession, alongside high inflation and increased unemployment (IFS, 2022; ONS, 2022).

Second is the wider confluence of continued macro forces occurring before the pandemic, including in the UK the withdrawal from the European Union, the spread of flexible and precarious working patterns (mainly in the Gig economy) and widespread unemployment and under‐employment amongst 16–24 year olds since the 2008 recession (O'Higgins, 2017). More recent issues have centred on living costs and wider geo‐political turbulence, potentially heightening the sense risk, life course and early career disruption of young people seeking to map out life trajectories. For graduates entering the labour market for the first time, the context of the COVID‐19 pandemic has some discernible immediate impacts, including ensuing recruitment freezing, training and internship withdrawal and increased take‐up for lower skilled non‐aligned employment with limited career prospects.

The economic context of the COVID‐19 pandemic therefore represents a significant challenge for graduates entering the labour market given the other contextual factors which may impact on the success and ease by which they can negotiate access to suitable jobs and move sustainably within the labour market. The one‐year period following graduation represents a significant transitionary period for HE graduates: it is a period where investment choices in HE need to translate into early career outcomes congruent with choices and related career expectations (Angeloni, 2021). The initial transition to the labour market is one which graduates must actively negotiate, apply some levels of agency and develop and present their identities as graduates to a range of important stakeholders, not least employers (Tomlinson et al., 2020; Tuononen et al., 2019). Early experiences of entering the labour market, including processes of further acquiring and then transferring knowledge, skills and forms of career capital are crucial to how successfully graduates can access sought‐after jobs. Depleted labour market conditions during this key transitional period potentially destabilise such formations, making it harder to map‐out early employment trajectories.

## 2 RECESSIONS, DISRUPTED GRADUATE TRANSITIONS AND EARLY CAREER SETBACKS

Economic recessions differentially impact different social and occupational groups, and overall, less qualified individuals are more likely to be adversely impacted than more qualified ones (Lewis & Heyes, 2020). The immediate effect of a recession is the freezing of vacancies and the relative retention of more established, often older, employers who carry lower training and on‐the‐job demands. Whilst the situation is generally better for HE graduates, it has been long observed that this group have experienced similar challenges over the past several decades. The current context of the COVID‐19 pandemic continues to evolve in new ways, but one of its immediate effects is to intensify a set of pre‐existing challenges in operation since the 2008 recession concerning graduates' integration into the labour market. Evidence also indicates income and opportunity dispersal across a very heterogeneous cohort of recent post‐2010 graduates (Green & Henseke, 2017; IFS, 2020), with some graduates more insulated from the effects of a weakened labour market or finding that their qualifications are still in demand from employers. The COVID‐19 pandemic downturn has increased UK graduate unemployment and overqualification is becoming far more widespread for university graduates (ONS, 2021).

The transitions from formal education to employment are generally understood to be marked by several features that are markedly different form previous periods when school and college leavers followed generally clear pathways into employment. These include more delayed and protected transition periods of entering a chosen employment field and achieving a clear role and identity during a crucial life stage (Wyn et al., 2020; Wolbers, 2013). Pathways into, and routes within, chosen fields are also more complex, often entailing periods of job mismatches, continued training and a postponement of the earnings and skills utilisation that graduates expected during university. Such processes are played out in contexts where the normative institutional structures of well‐defined occupational labour markets have been weakened by a combination of market volatility, job scarcity and employer commitment to training pathways (Walsh & Black, 2021).

The related concern is the effect of economic scarring for those entering employment for the first time (Scmillen & Umkehrer, 2017). Economic approaches to scarring have framed this problem mainly with respect to the subsequent adverse effects following from a period of initial unemployment. Accordingly, those who have experienced detrimental unemployment or under‐employment, are likely to experience future unemployment. This entails an adverse depreciation of human capital and skills gained from an initial qualification which become devalued over time, leading to subsequent earning loss and risk of future unemployment (Oreopoulus et al., 2012).

Alternative approaches to scarring, have tended to move beyond economic and earnings deficits and consider more socio‐psychological impacts (Breslin & Mustard, 2003). One of these concerns the impact this only has on health, well‐being and psychological functioning, and the ways people approach their future employment and conceive their job prospects. A potent and enduring form of employment scarring results from a lack of fulfilment of early career potential and a significant misalignment between a person's current and desired employment status. Employment scarring can further damage the way individuals think about their job futures and this can become a self‐fulfilling prophecy, including presenting oneself less favourably to employers. This extends to feelings of lower morale, weaker career efficacy and the destabilisation of early career goals and aspirations before entering the labour market. This may be self‐reinforcing if employers interpret a period of unemployment or under‐employment as signals of weaker employability potential.

This article explores the effect of the COVID‐19 pandemic on recent graduates' initial employment outcomes and how they experience the transition into a challenging economic context. The perceived effects of the COVID‐19 pandemic on first‐time graduate's entry to the labour market has been under‐researched, so this study sought to develop insights into how early entry graduates' early post‐graduation and early labour market transitions have been affected by pandemic‐affected labour market disruptions. This context is also significant given the lengthy periods of social restriction, increased digital interaction and, for 2020 graduates, rapid shift to online learning towards their final stages of study. The research aimed to understand the immediate impact of the pandemic on how graduates perceived their employment prospects, career progression and the potential latent adverse effects this has had on their longer‐term career planning and goals.

This research sought to investigate the impact of the COVID‐19 crisis on graduates' immediate and potentially longer‐term employment prospects and how they are adapting to this challenge. It examined recent graduates' perceptions of the effects of the COVID‐19 pandemic, including on their labour market entry, in order to provide further insight into the extent to which early employment scarring has occurred and its impacts on their early career trajectories and labour market integration. Whilst this context represents a specific timeframe and economic shock period, the study offers insights into early employment transitions during a challenging labour market context; and, given continued recessionary risks following a short period of stability, which could be experienced by future cohorts of graduates.

## 3 METHODS

The quantitative data are sourced from ‘The Impact of COVID‐19 on Recent Graduates’ Career Decisions and Outcomes' surveys. The surveys were longitudinal and investigated how UK university graduates fared in the transition from university to the job market during the COVID‐19 pandemic. The first survey, conducted between December 2020 and February 2021, captured the impact of the pandemic on graduate employment and how graduates felt about the employment process at roughly six months after graduation. The second survey was conducted in June through July 2021, at roughly a year after graduation, to measure the progress of graduates' progression to work. Both surveys were conducted by a research team at the Principal Investigator's institution with aid from the Association of Graduate Careers Advisory Services (AGCAS), after the successful competition of ethical approval from the lead institution. The data were collected by AGCAS using Smart Survey software and analysed using SPSS 28. AGCAS contacted careers service managers about the survey, and those managers invited new graduates from their intuitions to take part. The survey received 2767 useable responses out of the 2871 that had responded. The survey comprised both objective and subjective questions along with demographic questions. Just under a third of the participants were male, just under two thirds were female and under one per cent were non‐binary. Roughly 20% were from minority ethnic backgrounds. Over 60% of the participants were aged between 21 to 25 years old, representing the traditional age of graduates. Just under three quarters did not report a disability of any type. The second survey participants were those who had participated in the first survey and had indicated that they would be willing to take part in the second.

The participants were invited through email to participate. Again, the survey data were collected and analysed in the same format as the first. The response was 760 returned surveys, with 610 being completed and useable. Thus, there was a 22% response rate for this survey. There was not a statistically significant difference between those that participated in the first survey and those that took part in the second survey in gender, age, disability and national background. However, graduates who identify as White British were more likely to take part Survey 2. The proportion of the participants that identify as White British rose from 68.7% in Survey 1 to 74.8% in Survey 2. Nearly every other ethnic background decreased proportionally except Black British graduates, which rose slightly from .6 to .7% from Survey 1 to Survey 2.

The study allowed us to understand the interaction between graduates' perceptions of the current labour market, the perceived impacts of the COVID‐19 pandemic and other important dimensions, including well‐being, motivation and resilience. We were also able to gain insights into how early entry graduates' lives have been affected by related forms of social disruption brought about by lengthy periods of social restrictions, increasing digital interaction and the move towards homeworking. Many of these graduates had also experienced the rapid shift towards online learning during the very final stages of their studies and those who had found employment were likely to be socialised primarily into a homeworking environment.

The two surveys conducted as part of this research offered a significant opportunity to learn more about the employment situations, experiences and outcomes of graduates entering the labour market during the first stage of the COVID‐19 pandemic and up to 15 months following graduation, at a time when the social and economic context for school and university leavers remains uncertain. The longitudinal dimension enabled us to gather important insights into graduates' early employment progressions, real or perceived, and was often gleaned through the more qualitative elements of the research, including detailed open code survey responses and 80 individual interviews done over a nine‐month period in 2021. The interviews were conducted at two periods: the first wave between January and April 2021 involving 56 graduates who had completed the survey and a second wave between August and October 2021 involving 24 of the original 56 graduates.

## 4 KEY FINDINGS

### 4.1 Perceptions of the graduate labour market

The initial part of the survey addressed recent graduates' perceptions of the current labour market and its relationship to the pandemic environment. Table 1 indicates the main perceived impacts of the pandemic on graduates, whereby respondents were asked a series of questions to understand their perceptions of how the COVID‐19 pandemic affected the graduate jobs market and employment prospects. The data revealed significant perceived challenges in the current labour market at both a general and personal level. The vast majority of respondents perceived that the pandemic has had a detrimental impact on graduate employment prospects and also felt there to be declining opportunities through a fall in vacancies and job openings.

**TABLE 1:** Perceptions of the graduate labour market

Question	Mean	Strongly disagree	Disagree	Neither agree nor disagree	Agree	Strongly agree
I have found it a challenge to find graduate jobs I want to apply for	3.96	4.6%	7.6%	19.0%	24.9%	43.9%
I have noticed a fall in the number of available opportunities	4.13	2.3%	4.4%	19.5%	25.5%	48.3%
I have felt supported by employers during the recruitment process	2.64	16.1%	25.4%	40.8%	14.2%	3.5%
I have the sufficient experience that employers require	3.41	8.0%	18.2%	18.7%	35.2%	19.9%
I have found the recruitment process challenging	3.85	1.7%	7.0%	27.1%	32.9%	31.3%
I feel able to access different types of opportunities	2.76	13.2%	29.8%	29.7%	22.7%	4.7%

*Note*: *N* = 2767.

The survey revealed a number of perceived personal impacts on the ways recent graduates perceived their immediate employment outcomes and prospects. Significantly, the survey showed that the pandemic‐affected job market has resulted in a large proportion of graduates thinking differently about their futures (79.4%), facing greater challenges finding employment than they expected (71.9%). A large majority of participants either agreed (25.5%) or strongly agreed (48.3%) that they noticed a fall in the number of available opportunities.

Over two thirds agree (24.9%) or strongly agree (43.9%) that they have found it a challenge to find graduate jobs they want to apply for and agree (32.9%) or strongly agree (31.3%) that they have found the recruitment process challenging whilst few (17.7%) have felt supported by employers through the recruitment process.

### 4.2 Perceptions of the impacts of the COVID‐19 pandemic on graduate prospects

The data revealed that the current context is having an adverse impact on graduates' perceived early career prospects and related concerns about being able to develop pathways into relevant employment. In the period from either six to 18 months from graduation, a significant number of graduates reported struggling to find appropriate graduate‐level work, with the pandemic being attributed as a major challenge. Table 2 shows that the COVID‐19 pandemic has impacted graduates' ability to access the job market and had a negative impact on their career outlook. This revealed the difficulty graduates faced in entering the labour market during the pandemic, as slightly over half of the participants agreed (35.2%) or strongly agreed (19.9%) that they possessed the sufficient experience that employers require for a graduate role. These struggles are further reflected in Table 2 in that many graduates felt that COVID‐19 significantly damaged their job prospects (70.7%). Similarly, 72.7% either agreed or strongly agreed that COVID‐19 made them less confident about their future employment. These fears were coupled with many participants perceiving that the COVID‐19 pandemic resulted in greater challenges in finding employment than they expected. These perceived worries and difficulties in the recruitment process and the belief that there were fewer jobs on offer, led many participants to view the COVID‐19 pandemic had a detrimental impact on graduate employment prospects (83.6%).

**TABLE 2:** Perceived impact of COVID

Question	Mean	Strongly disagree	Disagree	Neither agree nor disagree	Agree	Strongly agree
COVID significantly damaged my job prospects	3.92	5.3%	11.5%	12.5%	27.5%	43.2%
COVID made me think differently about my future	4.06	2.8%	9.9%	7.8%	37.2%	42.2%
COVID affected the types of jobs I have targeted	3.75	5.3%	14.0%	16.7%	28.1%	35.8%
COVID made me less confident about my future employment	3.96	5.5%	12.3%	9.6%	26.5%	46.2%
COVID resulted in greater challenges finding employment than I expected	4.05	3.5%	8.1%	16.5%	24.0%	47.9%
COVID had a detrimental impact on graduates' employment prospects	4.29	1.5%	3.7%	11.3%	31.3%	52.3%

*Note*: *N* = 2767.

The open code survey responses and interviews confirmed the scale and prevalence of these challenges, as well as variance amongst the respondents in how much their prospects had been impacted by the pandemic. Many of the responses however revealed widespread concern and apprehension about both immediate and longer‐term outcomes with an overriding perception about the labour market being less accommodating. One of the immediate challenges was perceived to be the reduction in opportunities following vacancy shortages and employers scaling back workplaces and prioritising existing employees or those with the highest levels of experience.

There were widespread concerns that recent graduates were being misplaced or mismatched in the labour market, sometimes having to compete with non‐graduates or more experienced employees, or needing to undertake employment that was not aligned to expectations during their degree programmes. Many of these responses revealed the extent to which initial employment had either been withdrawn before they could embark or postponed entry and having to opt for non‐graduate work as a pecuniary measure and avoid risks of sustained unemployment.

Several key issues emerged when exploring the concerns raised. A prominent concern was over the perceived opportunity loss as a result of the pandemic environment they had entered on graduation, as well as the ongoing struggle to gain short‐term but potentially valuable work experience. This was especially felt amongst graduates who had not yet committed to a defined employment path but viewed the first year of graduation as a way of biding time and developing their emerging profile. This extended to perceived social relationship costs: concerns about reduced networking opportunities due to social confinement and the ability to form important in‐person relationships, including direct contact with key stakeholders or significant others who may be able to broker access to initial opportunities. Accessing opportunities for greater employer contact was perceived to be adversely impacted by the lockdown environment.

The interviews, as well as open survey responses, expressed a range of views on the COVID‐19 pandemic's direct and personal impact on employment prospects and how graduates appraised their situation. Those who felt the personal impacts less adversely tended to have secured employment not long after graduation, although even here there was awareness of a climate of greater insecurity for early career security and mobility. Overall, views range from more minimal impacts, typically amongst those who had embarked on more traditional and established pathways into the labour market, through to those who were frustrated but remaining optimistic about eventually securing graduate‐level employment. At the extreme end, although representing many of the views in both surveys and interviews, were those who conveyed discernible consternation at the current labour market and a clear sense of being marginalised from suitable opportunities.

However, many of the views on transitional barriers conveyed discernible disillusionment about the prospects for those leaving higher education:

The job market is an absolute train wreck at the moment. I reckon it'll be years until I get anywhere near a career that is satisfying, full‐time and gives appropriate work/life balance. (Female, Masters, Employment & Full‐time study, mid‐ranked Scottish HE)

I feel hopeless as a graduate right now and very low quite frankly, also employers and university make the process harder. Applying for jobs and getting no emails back is the most debilitating thing. (Female, BA, English Lit, Unemployed, Russell Group HE)

I have noticed that most companies are looking for experienced staff and even if it is entry level, the ones with experience get the job. I have mentioned in many a CV that I am willing to work for the minimum wage to gain the required experience. However, I suppose it is the inability of the companies to risk their staff to training new staff due to the virus. (Male, Creative Arts, BA, Other Such as travelling or taking care of someone Post‐92 HE)

The job prospects are abysmal, but it's about more than just that, this pandemic has changed my perspective in more personal, political, and philosophical ways. I hate the way that this world works and I do not want to be a part of it…In other words, I have given up on the idea that a typical “career” could ever sustain my life desires. (Male, BSc, Biological Sciences, Voluntary Employment, Post‐92 HE)

The ‘Catch 22’ work experience paradox was frequently referred to by many respondents with respect to being over‐qualified for many jobs yet lacking the experience to attain them, unless they had first‐time work experience. A proportion of graduates had continued part‐time work that they had undertaken since being in university to generate some income, mainly in lower‐skilled retail jobs. Yet the employability‐boosting potential of such experience as a way of enhancing marketability and signalling labour market adaptability, was perceived to diminish further into graduation. The majority were looking for more sustainable and aligned employment that would act as a catalyst to finding sustainable employment in targeted occupational fields:

It's a really tough industry because they put so much value on experience, but the thing is, most people can't afford to get the experience because you can only get the experience, generally, by working for free. And so many people can't afford to do that. They need to have weekend jobs to support themselves and they're studying full time. It's a sector that's biased more towards those who are more well‐off, generally. (Female, Marketing, Bsc, part‐time employed, Scottish HE)

Another notable finding was the lack of support which many graduates felt they were receiving from employers. The survey indicated that very few believed that they had been supported by employers, with further survey items revealing that they believed employers could support them in a number of key areas, including providing better information about available openings, providing internships for students before they graduate and providing more information about how they recruit graduates. The survey reported that the majority of respondents wanted more in the way of internship and training pathways, as well as better information about what they are looking for from graduates and how they recruit.

The more qualitative data indicated that many graduates perceived that more could be done to support pathways towards securing employment, particularly for those struggling to gain a foothold:

I've found time and time again employers will advertise for a graduate level job and then require 9–12 months experience, so you can't get the experience in order to get a job. It's a vicious cycle and it has detrimental effects on graduates and job opportunities. (Female, Architecture, Bsc, Freelance employment, English Post‐92 HE)

### 4.3 Impacts of unemployment and under‐employment

Over half of graduates have experienced being unemployed for longer than two weeks since March 2020 or have been employed in a job that did not draw on their graduate qualifications or skills, which has impacted their wellbeing and confidence and made many question the value of their degree. The average number of job applications made by graduates since March 2020 is 37, but ranges from zero to 1000, with seven graduates having made over 500 applications for graduate jobs since March 2020. Similarly, of the graduates who had applied for jobs since March 2020, just under a third did receive and accept a job offer, however a fifth of graduates only reached the CV/application form stage of the recruitment process.

The survey revealed a number of perceived adverse effects amongst those who had experienced a period of unemployment or under‐employment (Table 3). This suggests that, whilst still relatively early into graduation, initial scarring effects may be taking hold of a significant number of recently qualified graduates following unfavourable experiences of trying to enter the labour market. Over 80% of graduates who had experienced any of the negative employment outcomes report adverse impacts on their wellbeing. For three quarters of respondents, their experiences led to concerns that their skills were not going to be valued. Just under 70% felt that their experience had made them question the value of their degree, had negatively affected their employment outlook, or had affected their confidence about what they can offer to employers.

**TABLE 3:** Impact of unemployment and under‐employment from Survey 1

Question	Mean	Strongly disagree	Disagree	Neither agree nor disagree	Agree	Strongly agree
It negatively affected my outlook	4.05	3.5%	11.4%	15.0%	36.3%	33.0%
It felt like something that could become long‐term	3.89	6.2%	14.2%	14.2%	38.2%	23.7%
It affected my confidence about what I can offer employers	4.08	4.6%	13.2%	10.6%	31.5%	37.8%
It affected my well‐being and morale	4.35	7.1%	12.3%	8.0%	31.3%	48.9%
It made me concerned that my skills would not be valued	4.28	2.3&	9.8%	9.7%	29.0%	46.8%
It made me question the value of my degree	4.14	5.6%	13.8%	8.9%	22.8%	47.1%
It gave me the chance to develop experience and new skills	3.38	11.6%	26.3%	21.7%	29.3%	9.1%
It gave me the chance to reflect on what I want from my career	3.85	4.5%	13.2%	19.9%	41.2%	18.9%

*Note*: *N* = 2767.

There was widespread reporting in the open coded responses and interviews of the effects of sustained levels of unemployment or non‐aligned employment becoming longer‐term with the potential for being penalised for not being able to find more suitable initial employment. The issue of skill deprecation and diminished value of HE qualifications signals concerns over the lack of utility of human capital which was viewed to potentially penalise those whose positions were not improving over time. A related theme was the perceived competition with previous and future graduates whose early career prospects were not so adversely impacted as those graduating in either 2019 or 2020. Others expressed concerns about stigmatising effects and the potential negative signalling of being unemployed, especially compared to future graduates who may graduate during a growth period. This extended to concerns about potential stigma associated with graduating during a disruptive period of graduation, including the move to online exams and not being given a formal graduation.

Interviewed graduates were acutely aware of the rise in redundancies and furloughing since the first lockdown, which had impacted some who had been employed in part‐time work or who had seen their initial job or internship offers withdrawn. There were further concerns that recent graduates were being misplaced or mismatched in the labour market, sometimes having to compete with non‐graduates or more experienced employees, or undertaking employment that was not aligned to their degree programmes. Contingent stop‐gap plans such as developing further work experience or unpaid employment before progressing into securer graduate‐level jobs has become less viable for many. For some graduates, prolonged periods of unemployment had heightened the sense of urgency to get a job and sometimes resulted in them making applications that were not aligned to their targeted employment but as a short‐term measure to secure any kind of employment.

The data therefore reveal signs of initial scarring during the initial labour market entry phase not only in the forms of depletion in human capital and skills utilisation but also more social and psychological effects. These centred feelings of lowered confidence, reduced morale, career efficacy, fears of being less attractive to employers and corrosion to emerging identities that had formed before graduation. The adverse impacts on confidence and morale were mainly expressed as a less favourable reappraisal of being able to realise the career goals they had formed prior to graduating.

Experienced at its most intense, and over a sustained period, the evidence from the surveys and interviews indicated that a period of unemployment and job mismatch had destabilised graduates' career planning and goals and damaging their career confidence or perception of what is possible and how employable they may be. Amongst interviewees who were struggling to gain employment, some vividly described feelings of disillusionment, dejection and discouragement, particularly those who received multiple rejections. A number spoke very vividly of applying for similar jobs that their less‐qualified school‐leaving peers were undertaking, which had led them to question the value of their degree.

In a word, soul‐destroying. Made me seriously consider whether time invested in studying was worth it. (Female, Business Studies, Bsc part‐time employment, English HE)

Just bleak, depressing, wish I had not bothered. (Male, Computer Science, Bsc Unemployed, Scottish HE)

Graduates who had experienced sustained periods of unemployment described the lived realities of finding work, feeling in ‘limbo’ and ‘not knowing’ when suitable opportunities would arise. Whilst some graduates reported more intense feelings of disillusionment and isolation, others took reassurance from the wider context that had affected graduates at large. The framing of graduate unemployment as a systemic public problem within a recessionary economic climate helped in some cases mitigate the private dilemma of finding suitable graduate‐level employment and reduce attributions of personal failure. A distinction emerged between those in non‐graduate jobs who perceived this as a way of building up experience and those who were concerned that this could potentially damage longer‐term prospects and further distancing from suitable graduate jobs. The extent to which early employment experiences added value to their longer‐terms profiles depended on how much it served to build their skills and career resources beyond immediate financial imperative and could be used to signal attractiveness to employers.

### 4.4 Challenges further into graduating in the current labour market

The second wave survey provided an opportunity to assess graduates' career outcomes further into graduating. The follow‐up survey provided further insights into graduates' employment experiences and trajectories an additional six months on from graduation. Just under two thirds of the sample believed that their current employment was not aligned to their employment goals. Those who were unemployed or in jobs not aligned to their HE qualification were more likely to perceive being very far away from where they wanted to be and less likely to perceive that they had made meaningful progress. These graduates were also more likely to report that their current situation of being either unemployed or under‐employed had made them question the value of their degree, as well as concerns about the devaluing of the skills they could offer as graduate. Related were concerns about their skills not being valued or utilised in future employment.

Amongst the graduates who were unemployed or under‐employed at the time of the second survey, over 80% reported that this experience had made them very concerned that the skills they had acquired through HE were not going to be valued in the future. Nearly all unemployed graduates reported significant damage to morale, career motivation and confidence, as well as wider impact on well‐being and self‐esteem. Related, they were more likely to report deprecation to their skills and potential stigmatising effects of longer‐term unemployment.

Graduates reported a range of challenges in looking for and attaining their targeted employment. The most prominent challenge concerned staying focused and motivated, indicating that some of the above challenges are impacting graduates' ability to maintain their career goals. Another significant challenge reported was gaining the relevant work experience that employers require, a concern which those who were interviewed perceived to have been compounded by the decline in aligned work experience opportunities during the pandemic. Graduates also reported significant concern around their ability to develop meaningful and sustained social networks due to continued social restrictions. There was a perceived early career barrier for those who might not have been able to form significant career‐enhancing relationships during university or build upon initial contacts just before graduating. Others reported challenges around being able to market themselves and what they can offer, indicating perceived shortfalls in accessing early career opportunities that might enhance their marketability.

Six months further into graduation and approaching 15 months or longer from their initial graduation point, there are clear signs of adverse early career impacts and scarring amongst those who have been particularly impacted by the current climate. Many of these remain marginalised from and have experienced either longer‐term unemployment or being confined to jobs that are not utilising their existing human capital.

## 5 DISCUSSION, IMPLICATIONS AND CONCLUSIONS

This article has reported data on how graduates entering a very challenging labour market context perceive their career prospects and its impacts on their early employment outcomes, decisions and HE‐to‐work transitions. As a ‘shock event’ the pandemic context clearly disrupts what might be otherwise stabler and clear entry routes into the labour market and force individuals to re‐appraise their goals and anticipated outcomes (Akkermans et al., 2020). Many of those who were surveyed and interviewed perceived themselves as having the requisite skills, technical knowledge and profiles to find suitable graduate‐level employment. The need for continued resilience in a challenging job market environment was also reported widely. The overriding challenge was being able to exchange additional skills and human capital acquired during HE for sustainable initial employment and mapping out longer‐term strategic pathways to sought‐after career goals. A proportion of graduates in the study, albeit relatively small, had experienced smoother transitional pathways towards desired employment, although the experience for a significant amount of graduates in this study was one of delay, disorientation and reorientation from earlier formed career plans during higher education.

The study was undertaken at a profound moment of social and economic change which caused considerable consternation for those already in, and seeking to enter, the labour market. The adversities that began in early 2020 and which continued over the period of this study however cannot be seen in isolation: they instead followed ongoing disruptions in the youth and graduate market since the end of the 2000 s and we are now entering a further significant period of economic and geopolitical uncertainty which will continue to impact graduates' post‐university choices, progression and outcomes. The research period covered a specific, and notably challenging timeframe, but its findings have wider applicability to post‐HE transitions during weaker labour market contexts. Future research is required to track longer‐term labour market outcomes to better understand potentially longer‐terms effects of early employment penalties resulting from the COVID‐19 pandemic and how much initial scarring such as that reported in this study endures longer‐term. Research investigating transitions during precarious labour market contexts is important given the future labour market distributional challenges emanating from potential economic recession, related financial austerity and geo‐political instability impacting most national labour markets. Given the prediction that the UK is entering another significant period of recession (ONS, 2022), with the Bank of England suggesting economic shrinking beyond that of the COVID‐19 pandemic and the 2008 recession, this research highlights some of the key impacts this may have on those who graduate during a time of recession and economic uncertainty.

This research also raises implications for the continued support of graduate career development in HE contexts and early career stages. Discourses on so‐called Generation X tended to frame individuals born into the new millennium as empowered, independent and autonomous social agents who had internalised the new labour market realities of flexicurity, boundaryless career movements and entrepreneurial employment pursuits and content being unwedded to traditional career structures (Deloitte, 2018). The views and narratives recorded in this study indicate that this tends to overplay the propensity of recent graduates to achieve career and financial stability and job security, especially during the initial job entry phase. Many of this generation will have entered the labour market following the post‐2008 economic recession. Recent research has revealed how students and graduates within this cohort have internalised many of the new market realities including the need for flexibility, growing precariousness and short‐termness and a preparedness to manage their career planning accordingly (Jackson & Tomlinson, 2020). Such research also indicates increased levels of career proactivity and early engagement in career development which helps to mitigate the challenge of integrating. In the current study, whilst graduates appeared prepared to ‘bide time’ and ‘weigh up’ options, many found job entry uncertainty and the delayed time period from graduation unsettling and highly undesirable.

The survey and interviews revealed the scale of the challenges that recent graduates have faced since March 2020, even if they recorded a positive destination, such as full‐time employment or further study at the point of the first survey, they often still perceived being employed in a more precarious environment. Whilst there is clearly variance in the extent to which economic shock events such as the COVID‐19 pandemic have impacted recent transitions into an unstable labour market, the overall picture is one of considerable challenge with a perceived need for greater levels of early career development support, opportunity and training pathways.

The data reported a number of immediate impacts of trying to navigate a challenging labour market. First, this concerns how graduates respond to their immediate challenges and the evidence suggests a range of approaches to job searching, from more generic and speculative approaches through to more strategic. The open responses and interviews indicate that the former appeared to be based on a number of factors: first reflecting the anxiety to build financial capital and second to provide momentum at a time when early career progression had halted. This extended to perceptions of being against time and competing for a fresher cohort of graduates. Those taking strategic approaches tended to look for clear profile alignment and ensure better fit, including more openness to further building their profiles to enhance their suitability.

Secondly, the protracted period graduates experienced in entering employment of their choice appears to have a variety of effects. One is to reorientate to new career areas and consider further education and training. The other is to scale down earlier employment goals, continue with part‐time, non‐aligned employment as a means of maintaining some financial independence and wait for labour market opportunities to improve. The more qualitative dimension of this research indicated variability in early career narratives, mainly between those who had found employment relatively early and those who perceived they were gaining little progress and feeling notable anxiety. The latter group were likely to express acute feelings and of being timed‐out of the graduate entry‐level job market, competing with newer cohorts of graduates and that their skills and early work experiences were declining in value.

Thirdly, and related, there appears to be some deleterious impacts on those who have struggled to access employment or have remained in non‐graduate employment. These findings confirm potentially adverse impacts of enduring unemployment or under‐employment on early career outcomes and perspectives. The worst of these was adverse impacts on well‐being, health and motivation which was perceived to be self‐reinforcing if this prevented a graduate from continuing to build an early career narrative and result in disadvantageous signals about their potential job market value. It was notable that initial signs of scarring in this study had a more social and psychological dimension linked to well‐being, career planning and efficacy and identity disruption. Further, the data also reported widespread perceptions of being under‐supported during the crucial transition period, especially amongst employers who many felt could offer better training and support measures for those struggling to get a foothold into fist‐time employment.

The study raises several key equity issues in relation to the differential impacts of the current context and the capacity for different graduates to absorb these shocks. Those lacking traditional forms of capital, including economic capital, may find themselves less insulated from the strains of the current economic climate, not least because they may have less capacity and space to weigh up options and delay entry. As well as differences in their outcomes, there are also statistically significant differences in the resources that graduates can draw upon to support their transition into the labour market. Social capital is clearly important in facilitating better links between graduates' higher education and early employment experience and dependent on their ability to bridge weak ties and enabling social relations with significant others. Differences however exist in graduates' perceived social capital, with male graduates, nondisabled graduates and Black, African and Caribbean graduates all reporting greater confidence in their networking skills and connections with professionals in their chosen fields than female and non‐binary graduates, graduates with disabilities and those from other ethnic backgrounds.

The findings carry a range of policy‐related implications. One of these centres on mitigating the potentially damaging effects of entering a labour market during a challenging economic and socio‐political climate and finding ways of lessening their more adverse impacts. These work at both sides of the graduate labour market. At the supply level, and before graduates enter the market, graduating students need to be provided high‐quality forms of career provision and guidance that is effectively embedded into formal provision and made a stronger feature of learning experiences. This will require provision that encourages graduates to develop resources that better help them navigate declining opportunity structures, including enhancing their social capital and resilience, which may work towards buffering future scars of initial unemployment if they provide young people and those leaving formal education with a strategic pathway for managing sustainable employment (Bridgstock & Jackson, 2019). There is clearly a need for careers education, information, advice and guidance (CEIAG) and opportunities for work experience, experiential learning and skills development to be used in complementing existing formal curricula and embedded as part of institutions' employability ecosystem (AGCAS, 2022). A notable feature of this research is that many graduates reported needing careers support at the point of need and certainly in the twelve‐month period after graduation where additional guidance, support and mentoring was perceived to have some value in enhancing their career decisions and focus and minimise further disorientation. Given the impact of initial periods of unemployment and under‐employment, universities providing support for graduates beyond graduation may support recovery from or mitigation of scarring effects.

At the demand side, there is a clear need for coordinated employment policies, including investment in small to medium size enterprises (SMEs) and other training providers to enable smoother transitions for graduates struggling to find sustainable first‐time employment. More direct forms of employer engagement, which facilitate graduates' labour market entry, include structured forms of graduate traineeship or higher‐value kick‐start programmes that governments can subsidise, contingent on how well regulated and effective they are with respect to providing graduates with a pathway towards sustainable employment. These may be beneficial for graduates who have been unemployed for over six months and would benefit from structured forms of work‐integrated training within such a traineeship that could be used to build their career profile. Employers may also need to provide pathways for those with higher‐risk characteristics (including disabilities, special educational needs, mental health challenges) and others with less access to ‘protective’ structures that buffer the initial effects of unemployment.

One of the problematic paradoxes experienced by graduates in a recession is that of being over‐qualified yet inexperienced. During challenging transitional experiences, such as graduating straight into a pandemic context, this opens up a potential chasm whereby graduates are closed off from opportunities that may have some career building potential before they secure more sustainable positions. Employers may need to help plug opportunity losses and other experience gaps caused by the pandemic, given that those who have recently graduated into the pandemic and other recessionary period are at higher risk of employment penalties than those graduating in more favourable contexts. Additionally, careers services should continue to prioritise this in their relationships with employers, part of which involves embedding understanding of the ‘career journey’ during a student's time at university. A deeper dialogue with employers is important to facilitate understanding of how graduates ‘get in’ but also how they can ‘get on’. There needs to be further expansion of ideas around what type of policy options could reduce underemployment and longer‐term employment amongst the highly qualified and the extent to which skills are utilised in the labour force, rather than seeing this purely as a skills supply problem.

## CONFLICT OF INTEREST

The authors have no conflict of interests to declare.

## Data Availability

The data that support the findings of this study are openly available in The Impact of COVID‐10 on Recent Graduates' Career Decision at https://dx.doi.org/10.5255/UKDA‐SN‐855574.
